# Oleic acid stimulation of amino acid uptake in primary human trophoblast cells is mediated by phosphatidic acid and mTOR signaling

**DOI:** 10.1096/fba.2023-00113

**Published:** 2023-11-14

**Authors:** Elena Silva, Véronique Ferchaud‐Roucher, Anita Kramer, Lana Madi, Priyadarshini Pantham, Stephanie Chassen, Thomas Jansson, Theresa L. Powell

**Affiliations:** ^1^ Department of Obstetrics & Gynecology University of Colorado Anschutz Medical Campus Aurora Colorado USA; ^2^ University of Nantes‐INRAE UMR 1280 PhAN, CHU Nantes France; ^3^ Ob/Gyn & Reproductive Sciences University of California, San Diego La Jolla California USA; ^4^ Department of Pediatrics, Section of Neonatology University of Colorado, Anschutz Medical Campus Aurora Colorado USA

**Keywords:** fatty acids, human, Kennedy pathway, maternal‐fetal exchange, mechanistic target of rapamycin, phosphatidic acid, placenta, pregnancy

## Abstract

Normal fetal development is critically dependent on optimal nutrient supply by the placenta, and placental amino acid transport has been demonstrated to be positively associated with fetal growth. Mechanistic target of rapamycin (mTOR) is a positive regulator of placental amino acid transporters, such as System A. Oleic acid (OA) has been previously shown to have a stimulatory role on placental mTOR signaling and System A amino acid uptake in primary human trophoblast (PHT) cells. We investigated the mechanistic link between OA and System A activity in PHT. We found that inhibition of mTOR complex 1 or 2, using small interfering RNA to knock down raptor or rictor, prevented OA‐stimulated System A amino acid transport indicating the interaction of OA with mTOR. Phosphatidic acid (PA) is a key intermediary for phospholipid biosynthesis and a known regulator of the mTOR pathway; however, phospholipid biosynthetic pathways have not been extensively studied in placenta. We identified placental isoforms of acyl transferase enzymes involved in de novo phospholipid synthesis. Silencing of 1‐acylglycerol‐3‐phosphate‐O‐acyltransferase‐4, an enzyme in this pathway, prevented OA mediated stimulation of mTOR and System A amino acid transport. These data indicate that OA stimulates mTOR and amino acid transport in PHT cells mediated through de novo synthesis of PA. We speculate that fatty acids in the maternal circulation, such as OA, regulate placental functions critical for fetal growth by interaction with mTOR and that late pregnancy hyperlipidemia may be critical for increasing nutrient transfer to the fetus.

## Graphical abstract

Placental mechanistic target of rapamycin (mTOR) lipid sensing mechanism which modulates amino acid uptake. De novo synthesis of phosphatidic acid containing an oleic acid (18:1) acyl group stimulates placental mTOR. Activation of mTOR leads to increased System A mediated amino acid uptake.

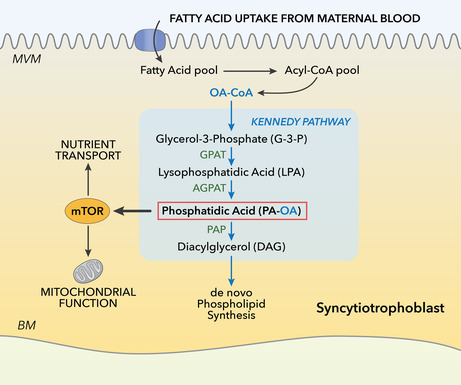

**Abbreviations**
AGPAT: 1‐acylglycerol‐3‐phosphate‐O‐acyltransferase
DHA: docosahexaenoic acid
GPAT: glycerol‐3‐phosphate acyltransferase
LC‐PUFA: long chain polyunsaturated fatty acids
mTOR: mechanistic target of rapamycin
OA: oleic acid
PA: phosphatidic acid
PHT: primary human trophoblast
PL: phospholipids
siRNA: small interfering RNA

## 1 INTRODUCTION

Fetal development and long‐term health outcomes of the child are critically dependent on normal placental function. For example, the capacity of the placenta to transport amino acids is positively associated with fetal growth in several pregnancy complications including obesity, diabetes, and intrauterine fetal growth restriction.^1^, ^2^, ^3^ We have reported that mechanistic target of rapamycin (mTOR) is a positive regulator of placental functions, including transport of amino acids,^4^ folate,^5^ and mitochondrial respiration.^6^ mTOR is a serine/threonine kinase that, in response to nutrients and growth factor signaling, regulates an array of cellular growth related processes such as protein translation, cytoskeleton reorganization, and cell migration.^7^, ^8^ mTOR exists as two distinct protein complexes in which the mTOR protein associates with regulatory‐associated protein of mTOR (raptor) to form mTORC1 or with rapamycin‐insensitive companion of mTOR (rictor) to form mTORC2. Both mTOR complexes are modulators of cell surface abundance of amino acid transporters in trophoblast cells mediated by distinct post translational mechanisms.^4^ For example, mTORC1 activation influences the plasma membrane trafficking of the specific amino acid transporter isoforms SLC38A2 (sodium dependent neutral amino acid transporter 2, a System A transporter isoform) and *SLC7A5* (LAT1, a System L transporter isoform) by regulating Nedd4‐2 mediated ubiquitination^9^ and mTORC2 regulates the actin skeleton responsible for insertion of amino acid transporters in the microvillous plasma membrane.^10^ Previous studies in cancer cell lines have demonstrated that mTOR also acts as a lipid sensor and phosphatidic acid (PA), a key intermediate of de novo phospholipid and triglyceride biosynthesis, is the form of lipid that modulates mTOR activity.^11^

During pregnancy, maternal fatty acids modulate an array of placental functions including development of extravillous trophoblast cells, lipid droplet formation, and amino acid uptake.^12^, ^13^, ^14^ Oleic acid (OA) (18:1 *n*‐9) is a predominant monounsaturated fatty acid in the maternal circulation, constituting 30% of the circulating nonesterified “free” fatty acid species during pregnancy. OA is known to have important effects on cellular functions in other cell types, including stimulation of fatty acid oxidation in skeletal muscle^15^ and prevention of insulin resistance and inflammation caused by saturated fatty acids in neuronal and muscle cells.^16^, ^17^ We have previously reported that OA stimulates mTOR signaling in primary human trophoblast (PHT) cells, whereas docosahexaenoic acid (DHA, 22:6 *n*‐3) inhibited mTOR.^18^ Cultured PHT cells, when exposed to a physiological mixture of four ^13^C uniformly labeled fatty acids species (palmitic acid, OA, linoleic acid, and DHA), rapidly accumulate these fatty acids in the cytoplasm and predominantly synthesize phospholipids (PL) rather than triglycerides or cholesterol esters,^19^ indicating robust de novo synthesis of PL in trophoblast cells. However, phospholipid biosynthesis and remodeling have not been robustly studied in human trophoblast.

While we have demonstrated that OA activates mTOR signaling, the mechanism linking OA to trophoblast mTOR signaling and subsequent regulation of amino acid transport remains unknown. PA, a key intermediate in the Kennedy pathway for de novo phospholipid biosynthesis, has been shown to be necessary for the stabilization and activation of mTOR complexes and that PA containing OA results in robust activation of mTOR in cancer cell lines.^11^ PA interacts with the rapamycin binding site to regulate mTOR activity.^20^, ^21^

The objective of the current study was to identify the mechanistic link between fatty acids, trophoblast mTOR signaling, and amino acid transport in order to better understand the relationship between maternal circulating lipids, placental function, and fetal growth. We tested the hypothesis that OA activates trophoblast amino acid transport mediated by PA synthesis and stimulation of mTOR. In addition, we identified the acyl transferase enzymes expressed in human placenta and used small interfering RNA (siRNA) mediated silencing approaches in PHT cell culture and physiologically relevant fatty acid concentrations to generate mechanistic data of high relevance to human pregnancy.

## 2 MATERIALS AND METHODS

### 2.1 Study subjects and tissue collection

Human placental tissue was collected from 15 term pregnancies (>37 weeks of gestation) following informed written consent at University of Colorado Anschutz Medical Campus. The “*n*” indicted for each experiment represents primary human cytotrophoblasts isolated from individual placentas. The study was approved by the Institutional Review Boards at the University of Colorado (14‐1073). Samples and relevant medical information were collected in the IRB‐approved data/tissue repository, and subsequently, study personnel were provided de‐identified samples and relevant clinical information used in this study (Table 1).

**TABLE 1:** Clinical data.

Maternal data	Mean ± SEM
*N*	15
Maternal age	31 ± 1.5
Race/ethnicity	3 White/Hispanic
	8 White/non‐Hispanic
	2 Black/non‐Hispanic
	1 unknown/Hispanic
	1 unknown/unknown
Body mass index	25.3 + 1.5
Delivery mode	7 NVSD
	8 C/S
Gestational age	39.5 + 0.2
*Neonate data*	
Neonate sex	9 Female
	6 Male
Birth weight	3501 g ± 92
Birth length	49.5 ± 0.6

### 2.2 Isolation and culture of PHTs

PHT cells were isolated from term placentas using the Kliman method^22^ involving DNAse/trypsin digestion and purification on a Percoll gradient as previously described.^12^ Cells were plated at a density of 2 x 10^6^ per well for amino acid uptake assays or 2.75 × 10^6^ cells per well in a 6‐well dish for siRNA gene silencing and subsequent experiments. Cells were cultured in equal volumes of Ham's F‐12 and high glucose DMEM, 10% fetal bovine serum (FBS; S11550, Atlanta Biologicals, Lawrenceville, GA), and antibiotics (penicillin, gentamicin and streptomycin) in 5% CO_2_, 95% atmospheric air at 37°C for 90 h. Culture media was changed daily.

Syncytialized PHT cells were treated with OA for 24 h starting at 66 h after plating. Culture media was changed to 1% FBS supplemented with bovine serum albumin (BSA, AK8909, Akron) in a 1:3 ratio with OA (100 μM, Sigma catalog # O1383). We have reported previously that this concentration of OA did not negatively affect PHT cell viability.^18^ PHT cells were studied at 90 h after plating.

### 2.3 System A and system L amino acid transport assay

Amino acid uptake in cultured PHT cells was determined at 90 h in culture. The activity of System A was assessed by measuring the Na^+^‐dependent uptake of [^14^C]methyl‐aminoisobutyric acid (MeAIB; 20 μm) and System L amino acid transporter activity was determined by 2‐amino‐2‐norbornane‐carboxylic acid (BCH; 64 μM)‐inhibitable uptake of [^3^H] leucine (0.0125 μm) for 8 min as described in detail previously.^23^ Total protein content in each well was determined by BCA assay (ThermoFisher Scientific, Waltham, MA), and the transport activity was expressed as pmol per mg of protein per minute (pmol/mg/min).

### 2.4 Western blotting

We used either automated capillary immunoblotting called JESS (Simple Western by ProteinSimple) or traditional poly‐acrylamide gel electrophoresis (PAGE) western blot to determine the abundance of de novo phospholipid synthesis enzymes. We ran the JESS plates based on the recommended manufacturer's settings (separation voltage of 375 for 25 min) with placental homogenates or PHT cells at a 1 ug/ul protein concentration per capillary. An equalizer sample was used between each plate to correct for variations, and human cerebellum (Novus Biologicals, catalog number NB820‐59180) and mouse brain were used as positive controls to confirm the presence of each target. Antibody details are found in Table 2

**TABLE 2:** Antibodies used in the study.

Name of antibody	Species monoclonal or polyclonal	Manufacturer and Catalog No.	Protein assay	Dilution used	IHC Dilution used
GPAT3	Rabbit; polyclonal	Sigma HPA029414	JESS	1:100	1:100
AGPAT2	Rabbit; polyclonal	Sigma HPA019544	JESS	1:100	1:50
AGPAT2	Rabbit; monoclonal	Cell Signaling CST14937	Western blot	1:1000	–
AGPAT4	Rabbit; polyclonal	Novus NBP1‐79870	Western blot	1:1000	1:750
S6 ribosomal protein	Rabbit; monoclonal	Call Signaling CST2217	Western blot	1:1000	–
Phospho‐S6 ribosomal protein (Ser235/236)	Rabbit; polyclonal	Cell Signaling CST2211	Western blot	1:1000	–

Abbreviation: AGPAT, 1‐acylglycerol‐3‐phosphate‐O‐acyltransferase.

Traditional PAGE western blot was performed as previously described^24^, ^25^ using a pre‐cast gel system (Bio‐rad). Twenty microgram total protein was loaded and separated on Bis‐Tris gels (4%–20%). Electrophoresis was performed at 120 V for 20 min then at a constant 150 V for 1 h. Proteins were transferred onto a PVDF membrane (Bio‐Rad Laboratories Inc.) overnight at 4°C at constant 35 V. The membrane was first stained for total protein with Amido Black Stain (Sigma‐Aldrich) and then cleared and blocked for 1 h in 5% BSA in TBS‐tween. Incubation with primary antibodies was carried out overnight at 4°C in 5% BSA/TBS‐tween or milk. The washed membrane was incubated for 1 h at room temperature with secondary antibody (peroxidase labeled anti‐Rabbit or anti mouse IgG, Cell Signaling Technology, Danvers, MA) diluted at 1:10000. Immunolabeling was made visible with SuperSignal West Pico Plus detection solution (Thermo Scientific) in a G:Box ChemiXL1.4 (SynGene, Cambridge, UK). Densitometry analysis of target protein bands was performed with GeneTools (SynGene), and target protein expression was adjusted for Amido Black to correct for loading.

PHT cells were collected in radioimmunoprecipitation assay buffer (ThermoFisher Scientific, Waltham, MA) containing phosphatase and protease inhibitors (Halt, ThermoFisher Scientific, Waltham, MA). PHT proteins were separated by electrophoresis, and protein expression was determined by western blot using commercial antibodies (Cell Signaling Technology, Boston, MA). Protein expression and phosphorylation of the downstream mTORC1 readout ribosomal protein S6 (rpS6K Ser235/236) and total ribosomal protein S6 were determined. Target band densities were normalized to total protein using Amido Black staining (Sigma‐Aldrich, St, Louis, MO). For each protein target, the mean density of the control sample bands was assigned an arbitrary value of 1 and all individual densitometry values were expressed relative to the control.

### 2.5 Immunohistochemistry acyl transferase and phospholipase enzymes

Immunohistochemistry was performed on term human placenta villous tissue (*n* = 3) as previously described.^26^ Briefly, tissue sections were fixed in 4% paraformaldehyde, embedded in paraffin blocks, and sectioned (5 μm). Tissue sections were fixed on slides and baked overnight at 37°C. Sections were blocked for 15 min with Thermo Scientific Pierce ABC kit buffer and blocking serum (catalog number 32054). Sections were probed with primary antibodies at dilutions listed in Table 2 and incubated overnight at 4°C. Negative controls were adjoining sections with primary antibody omission. Sections were then probed with provided secondary antibody (1:200 in ABC kit buffer) for 60 min at room temperature. Sections were assessed for antibody binding by incubation with the chromogen diaminobenzidine (SK‐4100, Vector Laboratories, Burlingame, CA) and counterstained with Mayer Hematoxylin (H‐3404, Vector Laboratories, Burlingame, CA).

### 2.6 RNA interference‐mediated silencing

We used RNA interference silencing targeting raptor (inhibits mTORC1) and rictor (inhibits mTORC2) and 1‐acylglycerol‐3‐phosphate‐O‐acyltransferase (AGPAT)‐4. Because levels of AGPAT‐2 are reported to be low in the placenta compared to AGPAT‐4,^27^ we opted to target AGPAT‐4 in the current experiments. Sigma Mission siRNA transfection reagent and small interference RNAs [Rictor (SASI_Hs02_00366683; 100 nM, OriGen Technologies siTran 2.0 siRNA Transfection reagent), Raptor (RAPTOR Human siRNA Oligo Duplex (locus ID 57521) 2 nmol) and AGPAT‐4 (SASI_Hs01_00228895, 100 nM)] were added to PHT cells after 18 h culture according to the manufacturer's recommendations. A non‐coding scrambled (SCR) sequence siRNA (SIC001, Sigma‐Aldrich) was also tested at the same concentration as the target sequence. After 24 h incubation, siRNAs were removed and fresh medium was added. At 90 h in culture, silencing efficiency was determined at the protein level using Western blot.

### 2.7 Data presentation and statistics

Data are presented as means +SEM. Each PHT isolation is from a unique placenta and cells were exposed to all treatments which allows us to use a paired analysis. The statistical significance of differences between control with and without OA treatment and siRNA mediated knockdown of AGPAT4 with and without OA treatment was assessed by repeated measures ANOVA with Tukey multiple comparisons post hoc test or paired *t*‐test using GraphPad Prism software (GraphPad Software Inc, San Diego, CA). Groups with unique letters are statistically significantly different, and a *p* value < 0.05 was considered significant.

## 3 RESULTS

### 3.1 Identification of Kennedy pathway acyl transferase and lands cycle enzymes in human placenta

**FIGURE 1:**
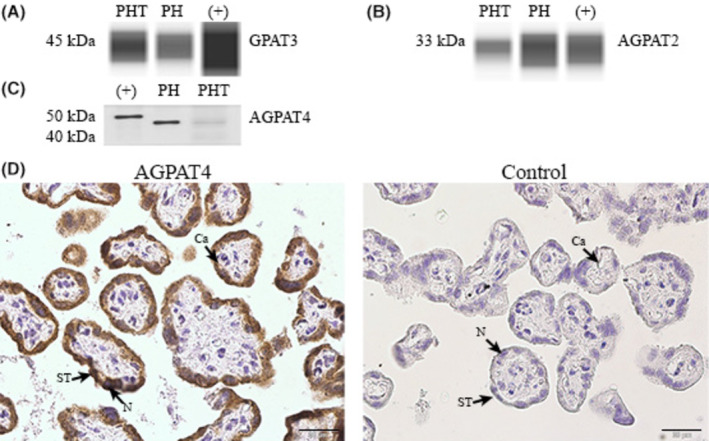
Expression of phospholipid biosynthesis enzymes in human placenta. Detection of acyl transferase enzymes in primary human trophoblast (PHT) cells and human placenta homogenate (PH), *n* = 3. Representative capillaries from Simple Western (JESS) used to determine the presence of the enzymes (A) glycerol phosphate acyl transferase isoform 3 (GPAT‐3), also known as 1‐acylglycerol‐3‐phosphate‐O‐acyltransferase (AGPAT)‐9 (1A, molecular weight 45 kDa) in placenta and human cerebellum as positive control. (B) Acylglycerol phosphate acyl transferase isoform2 (AGPAT‐2, also known as LPAAT‐β) (expected molecular weight 27 kDa) was found at 33 kD in placenta and human cerebellum. (C) Using traditional western blot, we detected AGPAT‐4 (expected MW 44 kDa) in placenta. Positive control samples for AGPAT4 were mouse brain. (D) Immunohistochemistry was used to show localization of AGPAT 4 isoform in human placenta syncytiotrophoblast, *n* = 3. Negative control was primary antibody omission. Key: N—nuclei, S—Syncytiotrophoblast, Ca—capillary.

We identified phospholipid de novo biosynthetic enzymes in human placental homogenates and cultured PHT and demonstrated the localization of these enzymes to the syncytiotrophoblast cell using immunohistochemistry (Figure 1). We identified GPAT‐3 as a band migrating at ~45 kDa in both term healthy human placental homogenates and cultured trophoblast cells using cerebellum (Novus Biologicals, catalog number NB820‐59180) as a positive control (Figure 1A). We identified protein expression of AGPAT‐2 in human placenta samples, at 33 kDa (Figure 1B). Likewise, we found that AGPAT‐4 migrated as a single band at approximately the expected MW of 45 kDa in human placenta samples with mouse brain as a positive control (Figure 1C). Differences in molecular weight appear to be organ‐specific changes in post translational modifications such as glycosylation or phosphorylation as we found similar differences with other positive controls. We found a strong signal for AGPAT4 in the syncytiotrophoblast using IHC (Figure 1D). These enzymes are critically important for de novo acyl transferase and the synthesis of PA and have not been extensively studied in human placenta.

### 3.2 mTOR signaling is required for OA‐stimulated PHT amino acid uptake

Confirming our previous reports,^18^ we found OA‐stimulated PHT cell System A but not System L transport activity. We silenced raptor (mTORC1) or rictor (mTORC2) expression in PHT (*n* = 4–7) using previously established siRNA methods and demonstrated a decrease in protein expression of raptor and rictor by 40%–50% (*p* < 0.05). As expected rictor knockdown inhibited trophoblast System A uptake but raptor inhibition failed to decrease System A activity in this series. This was inconsistent with previous results [9, 10] but we have no clear explanation for this difference. System L uptake was not impacted by either rictor or raptor knockdown. These results are found in the Figure S1A,B.

**FIGURE 2:**
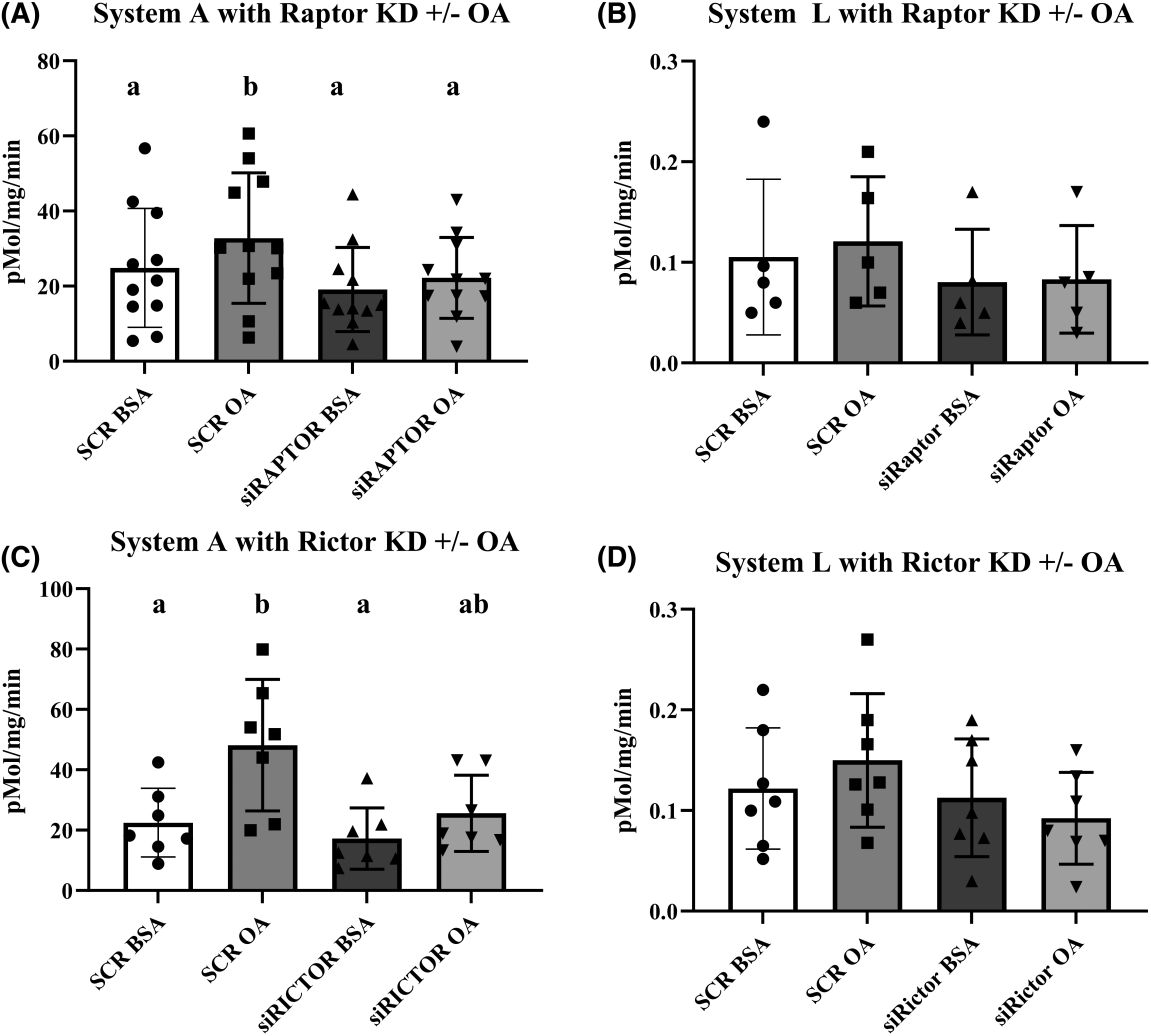
Raptor or rictor knockdown in primary human trophoblast (PHT) cells eliminates the impact of oleic acid (OA) on System A activity. PHT cells were transfected with small interfering (si) RNA targeting mTORC1 (raptor knock down) and mTORC2 (rictor knock down) or a non‐targeting scramble‐small interfering RNA (SCR). (A, C) System A amino acid uptake was measured by Na^+^ dependent uptake of [^14^C] methyl‐aminoisobutyric acid (MeAIB) in raptor and rictor knockdown compared to SCR, with and without stimulation with OA (OA 100 μM) or a fatty acid free BSA control, *n* = 7–8. (B, D) System L amino acid uptake, measured by BCH inhibitable 3H ‐Leucine uptake, was not different in any of the conditions tested (*n* = 5). Amino acid uptake (pMol/mg protein/minute) is represented as Mean + SEM. Repeated measures ANOVA and Tukey's multiple comparison post hoc test. Different letters denote significant differences at *p* < 0.05.

To characterize the mechanisms underlying the stimulation of System A amino acid uptake by OA in PHT cells, we tested whether mTOR signaling is required for OA stimulation of PHT amino acid uptake. Trophoblast cells treated with non‐coding (SCR) siRNA responded as expected with a significant increase in System A activity when exposed to OA. Knockdown of Raptor/mTORC1 prevented the increase in System A uptake in response to OA, suggesting that mTOR signaling is critically involved in OA stimulation of System A activity (Figure 2A, *n* = 11, repeated measures ANOVA, Tukey's post hoc comparison). Exposure to OA did not modulate System L activity in SCR or Raptor KD cells (Figure 2B, *n* = 5), in agreement with previous observations.^18^ Knockdown of Rictor/mTORC2 prevented the same level of increase in System A uptake in response to OA, suggesting that mTOR signaling is critically involved in OA stimulation of System A activity (Figure 2C, *n* = 7, repeated measures ANOVA, Tukey's post hoc comparison). Exposure to OA did not modulate System L activity SCR or Rictor KD cells (Figure 2B, *n* = ?) in agreement with previous observations.^18^

### 3.3 Involvement of AGPAT‐4 in OA stimulation of amino acid uptake

**FIGURE 3:**
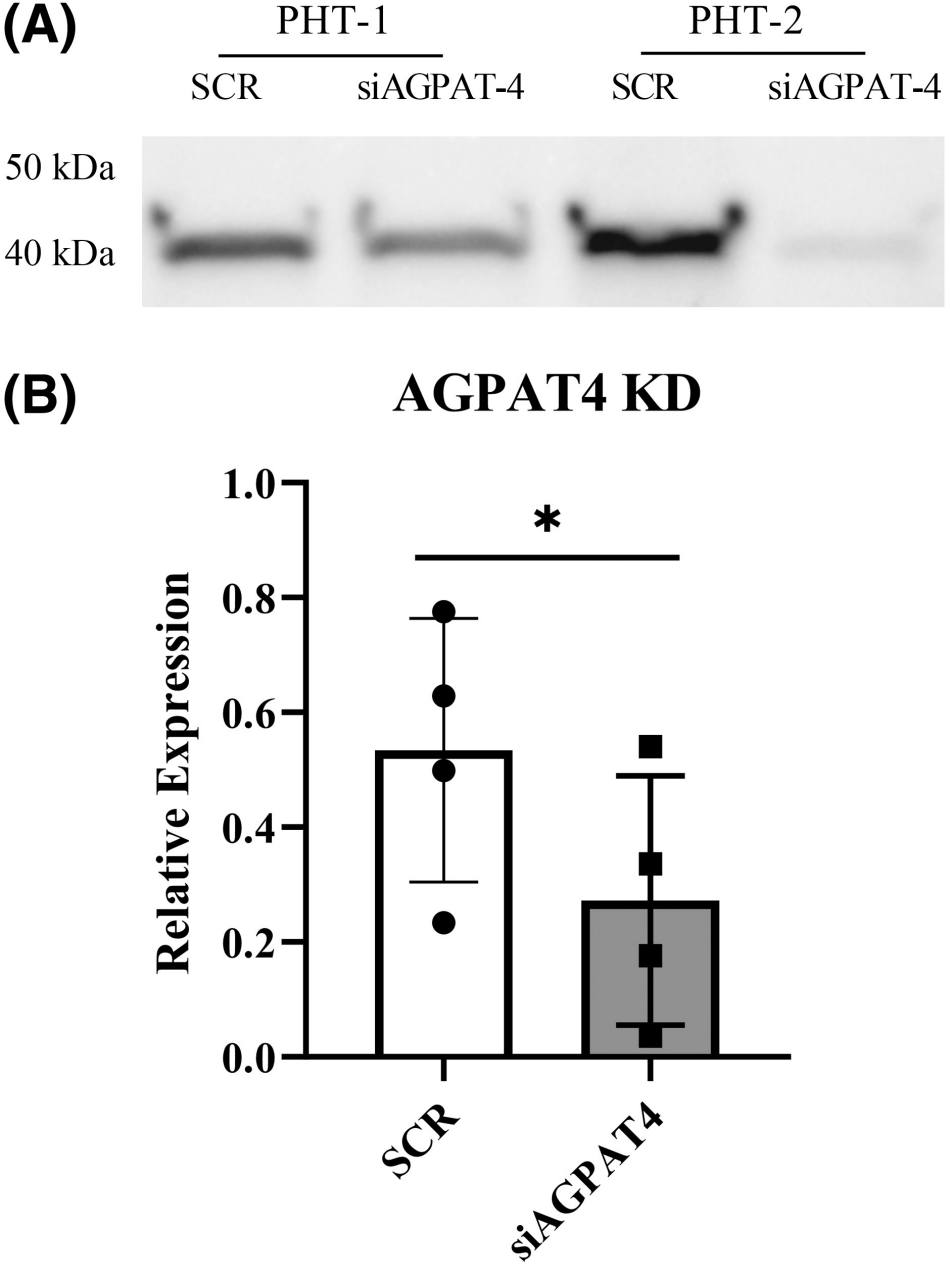
Knockdown of 1‐acylglycerol‐3‐phosphate‐O‐acyltransferase (AGPAT)4 in human primary trophoblast cells. Primary human trophoblast (PHT) cells were transfected with small interfering RNA targeting AGPAT‐4 (siAGPAT‐4) or a non‐targeting scramble‐small interfering RNA (siRNA) (SCR), *n* = 5. (A) Representative western blot image for AGPAT4 in PHT showing reduced expression at 90 h culture after siRNA exposure. (B) Transfection resulted in approximately 40% reduction in AGPAT4 expression, *n* = 5, one tailed *t*‐test. Asterisk denotes significant difference, *p* < 0.05.

**FIGURE 4:**
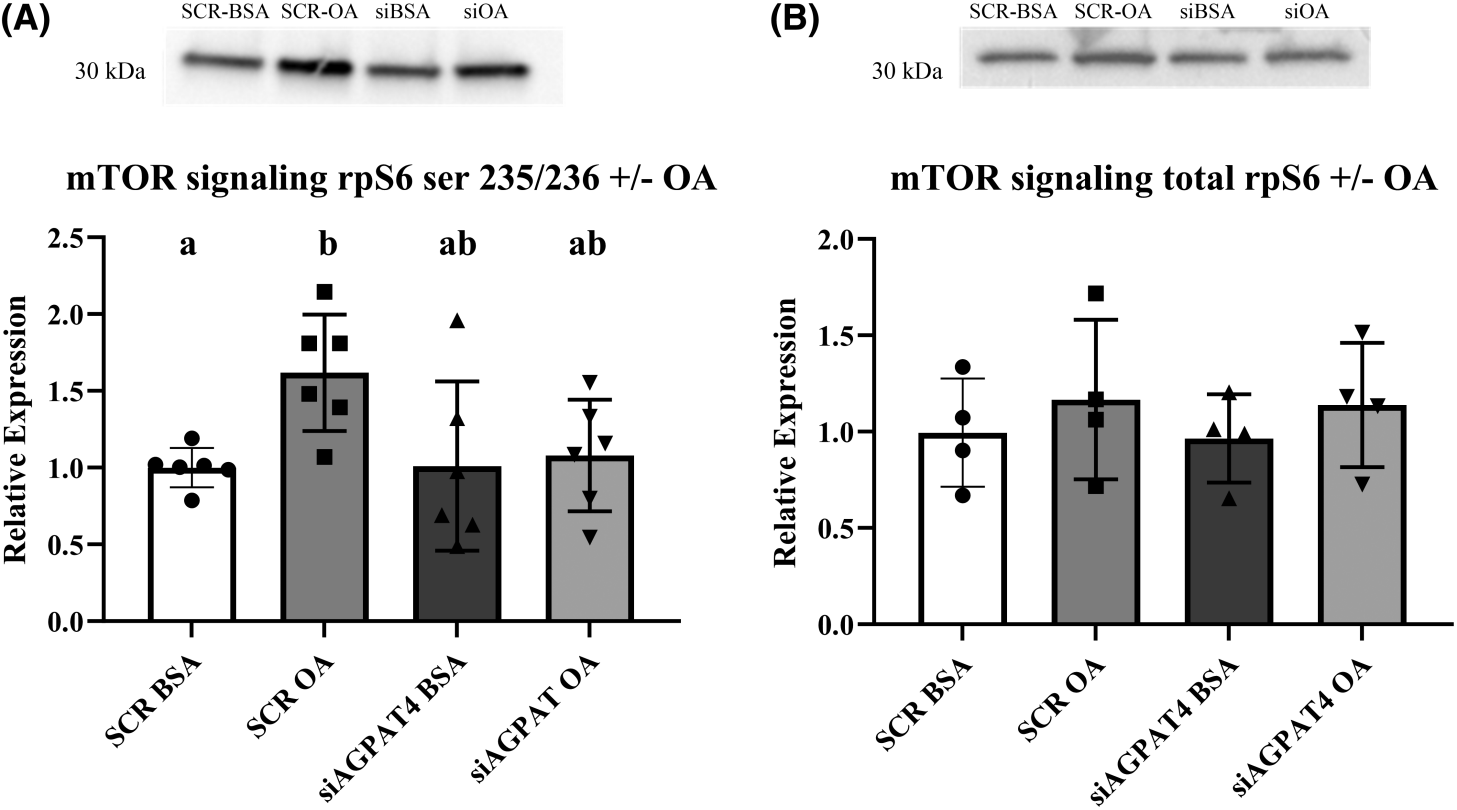
Impact of 1‐acylglycerol‐3‐phosphate‐O‐acyltransferase (AGPAT)‐4 silencing on mTOR signaling with and without oleic acid (OA) treatment. Primary human trophoblast cells were transfected with small interfering RNA targeting for AGPAT‐4 or a non‐targeting scramble‐small interfering RNA (SCR) and stimulated with OA (OA 100 μM). The expression of (A) phosphorylated (SER‐235) and (B) total ribosomal S‐6‐Kinase (rpS6) was measured by traditional western blot and is represented as Mean + SEM, *n* = 6, and analyzed by repeated measures ANOVA and Tukey's multiple comparison post hoc test. Different letters denote significant difference, *p* < 0.05.

**FIGURE 5:**
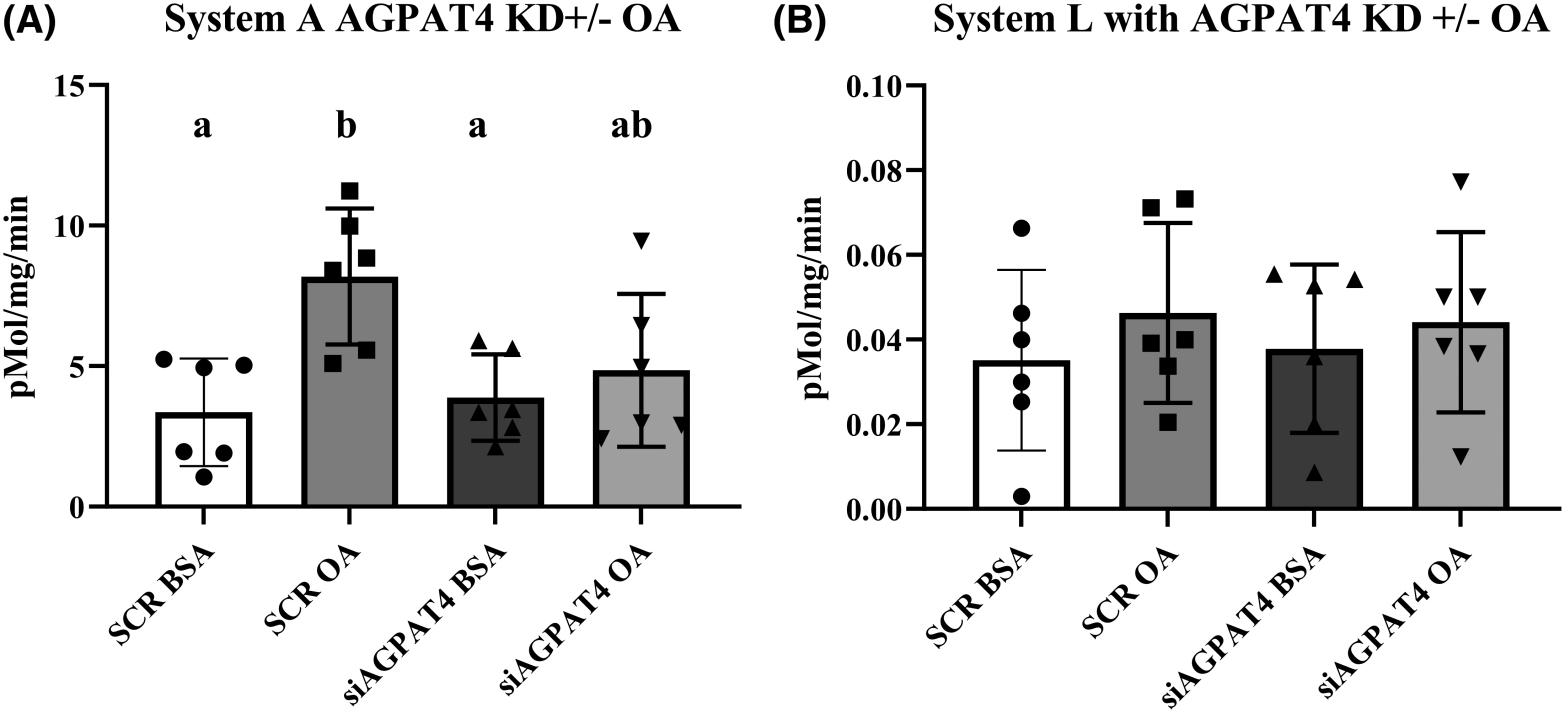
1‐acylglycerol‐3‐phosphate‐O‐acyltransferase (AGPAT)‐4 silencing prevented oleic acid (OA) stimulation of System A amino acid uptake. Primary human trophoblast cells were transfected with small interfering RNA targeting for AGPAT‐4 or a non‐targeting scramble‐small interfering RNA (SCR) and stimulated with OA (OA 100 μM). (A) System A amino acid activity measured by Na^+^ dependent uptake of [^14^C] methyl‐aminoisobutyric acid (MeAIB), *n* = 6. (B) System L amino acid uptake, measured by BCH inhibitable 3H ‐Leucine uptake, was not different in any of the conditions tested (*n* = 6). Data are represented as Mean + SEM and analyzed by repeated measures ANOVA with Tukey's multiple comparison post hoc test. Different letters denote significant difference, *p* < 0.05.

Silencing the AGPAT‐4 gene using siRNA in PHT, (*n* = 5) resulted in ~40% decrease in AGPAT‐4 protein expression (Figure 3, paired *t*‐est *p* = 0.04). Importantly, reducing expression of AGPAT4 did not change expression of AGPAT2 (data not shown). AGPAT4 silencing prevented the activation of mTOR by OA as evidenced by lack of increase in rpSK phosphorylation after OA treatment with AGPAT4 silencing (Figure 4A, *n* = 6, repeated measures ANOVA, Tukey's post hoc test). We see an increase in variability in the responsiveness with AGPAT4 KD so that the means were also not significantly different from the OA treated cells; however, there was no significant difference from untreated PHT cells as well. This is in line with a modest reduction in AGPAT4 expression of 40% seen in Figure 3. Total rpSK was not changed by silencing AGPAT4 or OA treatment (Figure 4B, *n* = 4). Importantly, reducing AGPAT4 expression reduced the OA stimulation of System A amino acid uptake (Figure 5A, *n* = 6 repeated measures ANOVA, Tukey's post hoc test), but had no impact on System L activity (Figure 5B, *n* = 6). Variation in response were noted which is in line with the 40% reduction in AGPAT4 expression. System A activity was not different from the control conditions but also lacked significance compared to the treated cells. The overall mean was reduced to near control levels suggesting transporter activity after OA exposure is due to a reduction in PA synthesis which represents a the mechanistic link between OA and mTOR regulation of amino acid transport in human placenta.

## 4 DISCUSSION

Our data support a role for OA stimulating amino acid transport in human placenta mediated by de novo synthesis of PA species containing OA. PA containing OA has been previously shown in cancer cell lines to be a potent activator of mTOR signaling,^11^, ^28^, ^29^ In human trophoblast, mTOR activation up‐regulates System A activity. This is a novel regulatory function of mTOR in human trophoblast indicating that availability of fatty acids for phospholipid and triglyceride synthesis is a critical nutrient sensing regulatory mechanism to support placental growth and function. To the best of our knowledge, this is the first report of a mechanistic link between OA, a highly abundant fatty acid in maternal circulation, and increased trophoblast PA synthesis, leading to activation of mTOR and up‐regulation of amino acid transport in the placenta. Our data demonstrate that mTOR in human placenta acts as a lipid sensor.

Previous work by our group has demonstrated that incubating PHT cells with OA at a physiological concentration of 100 μM for 24 h resulted in increased phosphorylation of mTOR pathway mediators, including mTOR, S6K1, and rpS6, as well as increased amino acid transport.^18^ Furthermore, PHT cells incubated in 50 μM DHA demonstrated reduced phosphorylation of p38MAPK, STAT3, mTOR, 4‐EBP1, and rpS6 and decreased amino acid transport, highlighting the opposing effects of these two distinct fatty acid species on trophoblast cellular signaling and function. We also demonstrated a negative correlation between placental DHA content and System A activity in a cohort of women receiving either 800 mg daily DHA supplementation or placebo,^30^ suggesting this is a biologically relevant mechanism for modulating amino acid uptake in human placenta.

De novo synthesis of PL is initiated by the addition of an acyl group to the sn‐1 position (carbon 1) of glycerol‐3‐phosphate, mediated by glycerol phosphate acyl transferase (GPAT) enzymes, to produce lysophosphatidic acid. Subsequently, the addition of a second acyl group to the sn‐2 position (carbon 2) of lysophosphatidic acid, mediated by acylglycerol phosphate acyl transferase (AGPAT) enzyme isoforms, results in the formation of PA.^31^, ^32^ PA, in addition to being a cellular signaling molecule, is an intermediate lipid in the formation of diacylglycerol which is the precursor for both triacylglycerols and PL.^32^ Overexpression of GPAT‐3 isoform (also known as LPPAT‐θ or AGPAT‐9) in the HEK‐293 cell line increases the incorporation of OA into PA and stimulates mTORC1.^33^, ^34^ GPAT‐3 is localized to the endoplasmic reticulum and the GPAT‐3 gene is expressed in kidney and adipose tissues with lower mRNA expression in the placenta.^34^ Previous data in cancer cells indicated that PA synthesis by AGPAT‐2 (also known as LPPAT‐β) constitutes a mechanistic link between OA and mTOR activation.^11^ Additionally, PA has been linked to increased intracellular amino acid levels in muscle cells of fish.^35^ AGPAT‐2 is a lipid sensor enzyme regulating mTOR^11^ and while AGPAT‐2 mRNA is abundant in adipose tissue, only low levels have been detected in the placenta.^27^ AGPAT‐4 (also known as LPAAT‐δ) is a mitochondrial acyltransferase that regulates synthesis of phosphatidylcholine and DHA incorporation in the brain^36^, ^37^ and is highly expressed at the mRNA level in the placenta,^27^ providing the rationale for focusing on AGPAT‐4 in the current study.

The enzymes associated with PA production and the role of PA in regulating cellular function have not been investigated in detail in human trophoblast cells. We have recently demonstrated that incubation of PHT with uniformly labeled ^13^C‐non esterified “free” fatty acids resulted in rapid incorporation into the cellular phospholipid fraction.^19^ This was true of all types of fatty acid tested: saturated, monounsaturated, essential, and long‐chain polyunsaturated fatty acids. We demonstrate here the expression of acyl transferase enzyme isoforms in the human placenta that are responsible for Kennedy pathway de novo synthesis of PA in trophoblast cells.

It has been previously reported that an increase in PA levels containing dipalmitoyl (16:0 palmitic acid) results in dissociation of rictor from the mTOR complex in hepatocytes.^38^ Palmitic acid treatment of PHT, at doses that did not cause elevation of caspase 3, had no impact on System A or phosphorylation of ribosomal S‐6 Kinase (rpS6)^12^, ^18^ in human trophoblast cells. An increase in PA after OA treatment has been shown to activate mTOR signaling in cancer cell lines and knockdown of AGPAT2 expression prevented the effect,^11^ suggesting that OA, when incorporated into PA, was a potent stimulator of mTOR activity. We have previously demonstrated that incubation of PHT in a physiological mixture of uniformly labeled ^13^C fatty acids resulted in rapid incorporation into PL^19^ suggesting robust de novo phospholipid biosynthesis in these cells through the Kennedy pathway.

We provide evidence that Kennedy pathway enzymes GPAT‐3, AGPAT‐2, and AGPAT‐4 are expressed at the protein level in PHT cells. To further support the specific involvement of the PA biosynthetic pathway in the stimulation of mTOR by OA, we used siRNA silencing approaches to downregulate the AGPAT4 isoform in the de novo synthesis pathway in cultured PHT cells. Our data demonstrated that reducing expression of AGPAT‐4 reduced OA stimulation of mTOR activity and amino acid transport. A limitation of this study is the lack of information on PA levels after AGPAT4 knockdown. Previous data have shown that PA is a regulator of mTOR via direct binding and stabilization of mTOR complex^20^, ^21^, ^29^ or activation of ERK 1/2 signaling pathway upstream of mTOR.^28^ We found no change in ERK phosphorylation in response to OA exposure or when AGPAT4 was silenced.

This is the first report of mTOR as a lipid sensing mechanism involving PA in the human placenta. These findings suggest a mechanistic role for specific maternal fatty acids to modulate placental PA levels and regulate mTOR signaling and placental functions such as amino acid transport. We propose mTOR regulation by specific fatty acids from the maternal circulation entering the syncytiotrophoblast and rapidly being incorporated into PL through the action of de novo biosynthetic enzymes. PL are particularly critical for the formation of the extensive microvillous plasma membrane which is in direct contact with maternal blood and serves as the interface for nutrient and oxygen uptake by the trophoblast cell. Lack of adequate fatty acids for robust phospholipid synthesis is part of the lipid sensing mechanism of mTOR. We show that this is through responsiveness to the intermediate signaling lipid, PA. Low cellular PA, especially containing OA, serves as an indication to reduce mTOR signaling and based on our previous experiments inhibition of placental mTOR will reduce transfer of nutrients to the fetus and slow intrauterine growth.^39^ Abundant maternal OA, as is seen in the second half of human pregnancy due to the well described hyperlipidemia of late gestation, would lead to activation of mTOR causing the stimulation of amino acid uptake and transfer to the fetus. Fatty acids may also regulate other trophoblast functions mediated by mTOR signaling. For example, we recently reported that mTORC1, but not mTORC2, is a positive regulator of trophoblast oxidative phosphorylation mediated by effects on mitochondrial biogenesis.^6^ Thus, by controlling syncytiotrophoblast ATP production, changes in fatty acid levels may modulate all energy‐requiring processes including active transport and protein synthesis mediated by mTOR signaling.

A major physiologic response to pregnancy is a profound increase in circulating lipids; for example triglycerides increase ~200% from early to late pregnancy.^40^, ^41^, ^42^ Dietary fat content will be reflected in triglycerides in the maternal circulation,^43^ and OA is a highly abundant fatty acid in western diets. These data suggest that elevation of maternal lipids in the second half of pregnancy may stimulate nutrient delivery of both lipids and amino acids to the fetus and potentially regulate other vital functions of the placenta. In general agreement with this concept, maternal obesity has been reported to increase OA uptake by placentas of female fetuses^44^ and is associated with fetal overgrowth.

We conclude from these findings that enzymes responsible for phospholipid and triglyceride biosynthesis are present in human trophoblast and that OA stimulates amino acid transport in trophoblast cells mediated by the synthesis of PA species and their activation of mTOR signaling. These findings highlight a novel and important role of a highly abundant fatty acid, OA, in regulating trophoblast function. Our previous studies suggest that high dietary long chain PUFAs, in particular *n*‐3 DHA, may have an opposing effect and counterbalance the stimulation by OA. Circulating fatty acid levels are dependent on dietary intake, particularly for less abundant species such as *n*‐3 DHA; therefore, it is plausible that modifications in maternal dietary fat consumption impact placental function. These data taken together suggest that maternal lipid status and dietary lipid composition including the naturally occurring hyperlipidemia of pregnancy impact the placental amino acid transport capacity mediated by trophoblast mTOR signaling, and play a critical role in fetal growth and development.

## AUTHOR CONTRIBUTORS

Elena Silva, Lana Madi, Anita Kramer, and Priyadarshini Pantham conducted the experiments. All authors conceived the experiments, participated in development of the project, and reviewed the manuscript.

## FUNDING INFORMATION

This study was supported by grants from NIH (HD104644 to TLP and HD068370 to TJ) and by NIH/NCATS Colorado CTSA Grant Number UL1 TR001082. Contents are the authors' sole responsibility and do not necessarily represent official NIH views.

## DISCLOSURES

The authors have no competing interests to declare.

## ETHICS STATEMENT

Collection of human placentas at the time of term pregnancy delivery was conducted after informed signed consent and was approved by the Institutional Review Boards at the University of Colorado (14‐1073).

## Supporting information


Figure S1.

## Data Availability

All data needed to evaluate the conclusions of this paper are presented in the paper or supplementary materials. Additional information or data can be requested from the corresponding author.
