# Supplementary figures and images for: Oleic acid stimulation of amino acid uptake in primary human trophoblast cells is mediated by phosphatidic acid and mTOR signaling
Source: FASEB Bioadv. 2023 Nov 14;6(1):1–11. doi: 10.1096/fba.2023-00113 (PMC10782470; doi:10.1096/fba.2023-00113)

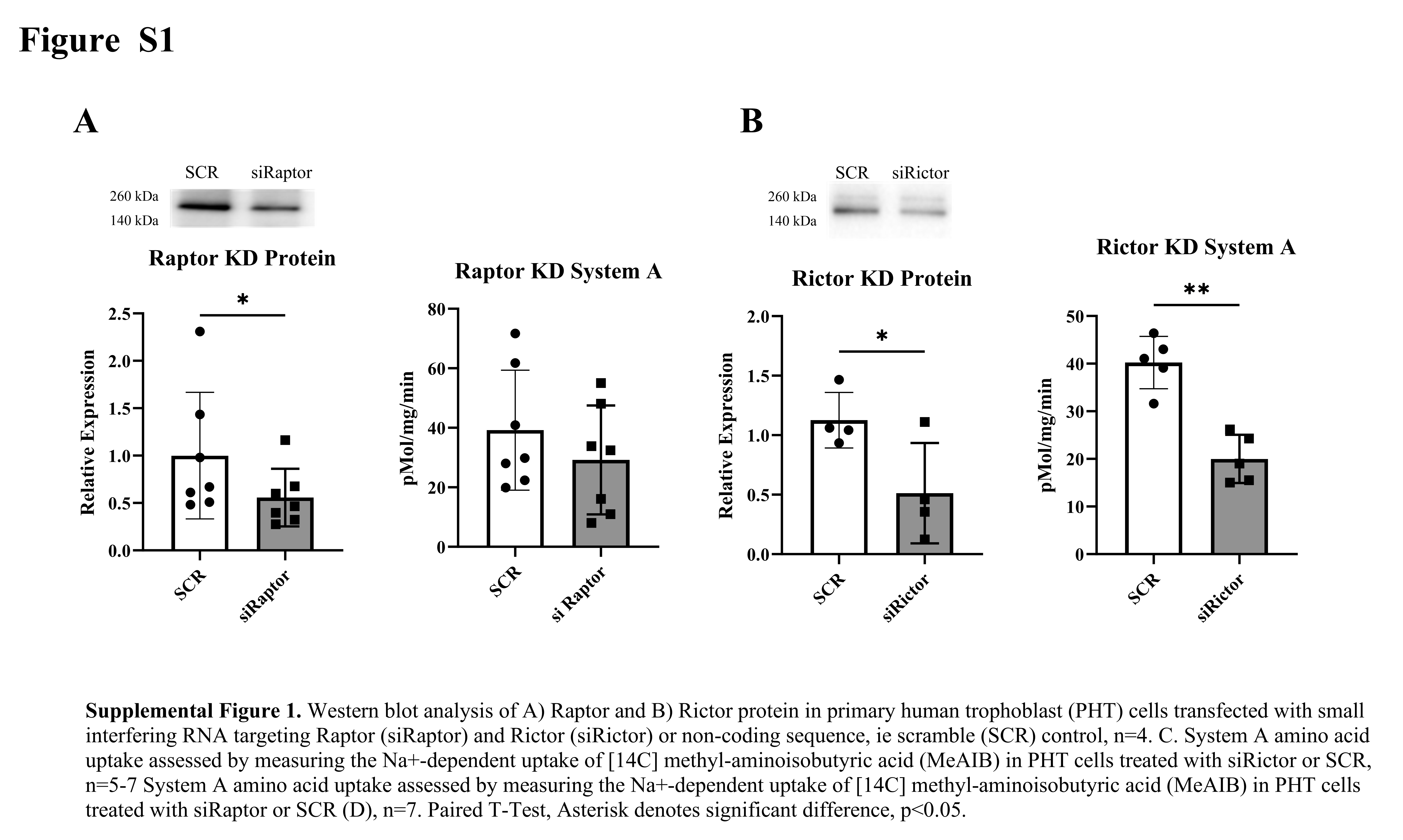

Supplement: Supplementary file 1 — Figure S1. [file FBA2-6-1-s001.tif]
